# Nomograms predicting survival and patterns of failure in patients with cervical cancer treated with concurrent chemoradiotherapy: A special focus on lymph nodes metastases

**DOI:** 10.1371/journal.pone.0214498

**Published:** 2019-04-15

**Authors:** Weiping Wang, Xiaoliang Liu, Qingyu Meng, Fuquan Zhang, Ke Hu

**Affiliations:** Department of Radiation Oncology, Peking Union Medical College Hospital, Chinese Academy of Medical Sciences & Peking Union Medical College, Beijing, People’s Republic of China; Tata Memorial Centre, INDIA

## Abstract

**Objective:**

To construct nomograms predicting survival and patterns of failure in patients with cervical cancer treated with concurrent chemoradiotherapy (CCRT).

**Methods:**

A total of 833 patients with cervical cancer treated with definitive radiotherapy or CCRT in our institute from January 2011 to December 2014 were included. Cox proportional hazard regression models were used in univariate and multivariate analysis. The following variables were included in the univariate analysis: histology, FIGO stage, lymph node metastases (para-aortic, pelvic, common iliac, binary pelvic, and binary common iliac LNMs), the number of pelvic metastatic lymph nodes (MLNs), and the diameter of pelvic MLNs. Nomograms predicting the 3- and 5-year overall survival (OS), disease-free survival (DFS), local control (LC) and distant metastasis-free (DMF) were constructed. The nomograms were internally validated with respect to discrimination and calibration.

**Results:**

The median follow-up period was 36.4 months (range,1.0 to 76.2 months). After univariate and multivariate analysis, histology, FIGO stage, para-aortic LNM, pelvic LNM, number of MLNs and diameter of pelvic MLNs significantly predicted OS, DFS, LC or DMF. Nomograms predicting the 3- and 5-year OS, DFS, LC and DMF were constructed incorporating these significant variables. These nomograms showed good discrimination and calibration, with a concordance index of 0.73 for predicting OS, 0.71 for DFS, 0.73 for LC and 0.67 for DMF.

**Conclusion:**

We constructed nomograms predicting survival and patterns of failure with a special focus on regional LNM in patients with cervical cancer treated with concurrent chemoradiotherapy.

## Introduction

Currently, the standard treatment approach for locally advanced cervical cancer is concurrent chemoradiotherapy (CCRT) [1–3]. Although the use of concurrent chemotherapy has significantly improved the survival of patients with cervical cancer [1–3], 24.9% to 43% of patients experienced tumor relapse after treatment[1, 4, 5]. If we can accurately identify the patients with a high risk of tumor relapse, and give them more aggressive treatment, we will further increase the survival of patients with cervical cancer.

In general, patients with advanced stage cancer have a higher risk of tumor relapse. The International Federation of Gynecology and Obstetrics (FIGO) staging system [6] is the most widely used staging system for cervical cancer. It is based on anatomic-compartment spread of cervical cancer and is effective for evaluating surgical resection. Regional lymph node metastases (LNM) are another important prognostic factor for patients with cervical cancer [7–11]. Because cervical cancer is most prevalent in developing countries with limited imaging resource, FIGO staging system dose not include LNM. As a result, the FIGO staging system is not accurate enough to predict the prognosis, with a concordance index (c-index) less than 0.6 [8, 10].

Regional LNM is incorporated into the American Joint Committee on Cancer (AJCC) staging system for cervical cancer. For some other tumors, N stage is subclassified with different lymph node regions (lung cancer, corpus uteri carcinoma etc), the number of metastatic lymph nodes (MLNs), (colorectal cancer, esophageal cancer etc.), the diameter of MLNs (vulva cancer, larynx cancer etc), ipsilateral/bilateral LNM (nasophagus cancer, thyroid cancer etc), or a combination of them in the AJCC staging system. However, for cervical cancer, N stage is only classified into with or without regional LNM in the AJCC staging system.

Nomograms have been used in the treatment of many malignancies in recent years, including patients with cervical cancer treated with definitive CCRT or radiotherapy [7–11]. However, the previous studies paid little attention to the subclassification of regional LNM. In the present study, we constructed a nomogram with a special focus on LNM, in which regional LNM were subdivided by region, as ipsilateral/bilateral, and number and diameter.

## Materials and methods

### Patients

The Institutional Review Board (IRB) of Peking Union Medical College Hospital reviewed and approved the protocol. The ethical committee process number is B251. The consent was not necessary, because patient records and information were anonymized and de-identified prior to analysis. Participants were recruited to the study from March 18th, 2018. Patients with cervical cancer who received definitive radiotherapy or CCRT in our institute between January 2011 and December 2014 were included. The inclusive criteria were as follows: histologically confirmed cervical cancer; FIGO stage IB1-IVA; no evidence of distant metastases; and treated with definitive radiotherapy or CCRT. Patients who had received previous surgery or radiotherapy were excluded. Finally, a total of 833 patients were used to construct the nomograms.

### Treatment

As described previously [12, 13], a dose of 50.4 Gy in 28 fractions was delivered to the pelvis with intensity-modulated radiation therapy or helical tomotherapy. Patients with regional LNM received dose-escalated radiotherapy, with 59–61 Gy prescribed to the involved lymph nodes. Weekly cone-beam CT/CT-on-rail or daily megavolt CT were used for image guidance. A dose of 30–36 Gy was prescribed to point A with high-dose-rate intracavitary brachytherapy.

Cisplatin (40 mg/m^2^/week) was used in concurrent chemotherapy. Paclitaxel (60–80 mg/m^2^/week) was administered to patients with renal dysfunction.

### Follow-up evaluation

Patients received gynecological examination and pelvic magnetic resonance imaging (MRI)/CT one month after chemoradiotherapy. After that, follow-up examinations were performed every three months during the first two years, every six months during three to five years, and once a year thereafter.

### Variables and statistics

The endpoints of this study were overall survival (OS), disease-free survival (DFS), local control (LC) and distant metastasis-free (DMF). Clinical data were collected from the medical records. To identify variables predicting OS, DFS, LC and DMF, the following variables were included: histology, FIGO stage, LMNs (para-aortic, pelvic, common iliac, bilateral pelvic LNM, and bilateral common iliac LNMs), number of pelvic MLNs, and diameter of pelvic MLNs. All variables were considered as categorical variables. Histology was classified as squamous cell carcinoma (SCC) or non-squamous cell carcinoma (non-SCC). FIGO stage was categorized into 3 groups: stage IB, stage IIA-IIB, and stage IIIA-IVA. In our institute, lymphadenectomy was not a routine procedure for cervical cancer patients receiving definitive chemoradiotherapy. Regional LNM were diagnosed with imaging modalities in this study. Regional lymph nodes confirmed by positron emission tomography/computed tomography (PET/CT), or with short axis diameter longer than 1cm on CT, were defined as MLNs[14]. As described previously [15], the para-aortic lymph node region was defined as the area adjacent to the aorta or inferior vena cava from the top T11 to the lower aortic bifurcation. The common iliac lymph node region was defined as the area adjacent to the common iliac vessels from the aortic bifurcation to the division of the common iliac artery. The number of pelvic MLNs were categorized into < 4 and ≥ 4. The diameter of the MLNs was the short axis diameter of the largest lymph node and patients were categorized in two groups with pelvic MLNs < 2cm or ≥ 2cm.

The OS, DFS, LC and DMF rates were estimated with the Kaplan-Meier method. The Cox regression model was used in univariate and multivariate analysis. First, the bivariate relationship between OS/DFS and variables was assessed. Next, the significant predictive variables obtained by univariate analyses were tested by multivariable Cox proportional hazards regression models. As described previously [16], the nomogram was built based on the results of Cox regression. The model was internally validated with respect to discrimination and calibration. Discrimination was assessed by the c-index, which estimates the probability of concordance between predicted and observed responses. The bootstrap method was used to obtain a relatively unbiased estimate. Calibration was carried out by comparing the mean of the nomogram-calculated survival with the mean survival observed by the Kaplan-Meier method. All analyses were performed using R version 3.0.0 (http://cran.rproject.org/mirrors.html) and SPSS (version 22.0). A two-sided p value <0.05 was considered significant.

## Results

The demographic, clinical, and treatment characteristics of the 833 enrolled patients are shown in Table 1. The median follow-up was 36.4 months (range,1.0 to 76.2 months). During follow-up, 195 patients (23.4%) experienced tumor relapse, including 74 patients with local recurrence, 95 patients with distant metastasis, and 26 patients with concurrent local recurrence and distant metastasis. The 3-year OS, DFS, LC and DMF rates were 84.0%, 75.5%, 87.5% and 84.9%, respectively. The estimated 5-year OS, DFS, LC and DMF rates were 77.7%, 71.7%, 87.1% and 82.5%, respectively.

**Table 1:** The characteristics of the patients.

Characteristics	n	Percentage (%)
Age (y)		
Median	51 (range, 23–88)	
<65	742	89.1%
≥65	91	10.9%
Histology		
SCC	744	89.3%
Adenocarcinoma	71	8.5%
ASC	9	1.1%
Others	9	1.1%
FIGO stage		
IB	102	12.2%
IIA	73	8.8%
IIB	500	60.0%
IIIA	28	3.4%
IIIB	126	15.1%
IVA	4	0.5%
LNM		
Regional LNM	238	28.6%
Para-aortic LNM	55	6.6%
Pelvic LNM	233	28.0%
Common iliac LNM	62	7.4%
Bilateral pelvic LNM	135	16.2%
Bilateral common iliac LNM	16	1.9%
Number of pelvic MLNs		
1–3	191	22.9%
≥4	42	5.0%
Size of pelvic MLNs		
Median	1.4cm (range, 0.7–4.5 cm)	
<2 cm	176	21.1%
≥2 cm	57	6.8%
Concurrent chemotherapy		
No	145	17.4%
1–3 cycles	103	12.3%
≥4 cycles	585	70.2%

Abbreviations: ASC = adenosquamous cell carcinoma; FIGO = International Federation of Gynecology and Obstetrics; LNM = lymph nodes metastases; MLNs = metastatic lymph nodes; non-SCC = non-squamous cell carcinoma; SCC = squamous cell carcinoma.

**Fig 1:**
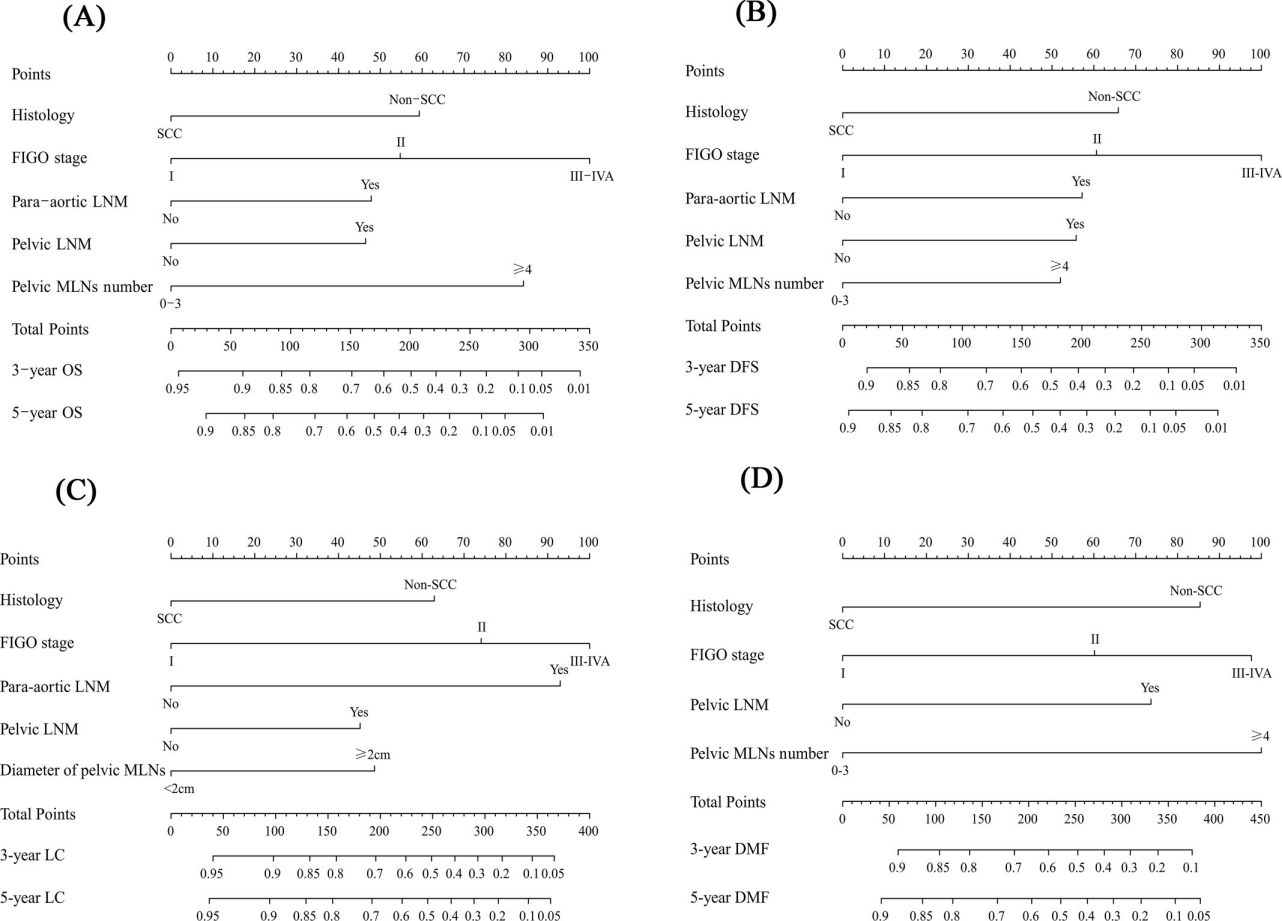
**Nomogram for determining the 3- and 5-year overall survival (OS, Fig 1A), disease-free survival (DFS, Fig 1B), local control (LC, Fig 1C) and distant metastasis-free (DMF, Fig 1D) of patients with cervical cancer treated with chemoradiotherapy.** FIGO = International Federation of Gynecology and Obstetrics; LNM = lymph nodes metastases; MLNs = metastatic lymph nodes; non-SCC = non-squamous cell carcinoma; SCC = squamous cell carcinoma.

Univariate analysis showed that nine variables, including histology, FIGO stage, para-aortic LNM, pelvic LNM, common iliac LNM, bilateral pelvic LNM, bilateral common iliac LNM, number of pelvic MLNs and diameter of pelvic MLNs, were prognostic factors of both OS, DFS, LC and DMF. After multivariate analysis, histology, FIGO stage and pelvic LNM maintained their significance in predicting OS, DFS, LC and DMF. Para-aortic LNM is an independent factor of OS, DFS and LC. Number of pelvic MLNs in an independent predictor of OS, DFS and DMF. And diameter of pelvic MLNs significantly predicted LC. Detailed data of univariate and multivariate analysis for OS, DFS, LC and DMF are shown in Tables 2–5. Nomograms for predicting 3- and 5-year OS (Fig 1A), DFS (Fig 1B), LC (Fig 1C) and DMF (Fig 1D) rates were constructed using these variables.

**Table 2:** Univariate and multivariate analysis for overall survival.

Variables	Univariate analysis		Multivariate analysis	
	HR (95%CI)	P	HR (95%CI)	p
Histology				
SCC	Reference		Reference	
Non-SCC	2.06 (1.33–3.20)	0.001	2.30 (1.46–3.63)	<0.001
FIGO stage				
IB	Reference		Reference	
IIA, IIB	2.73 (1.20–6.24)	0.017	2.02 (0.88–4.66)	0.097
IIIA, IIIB, IVA	5.64 (2.40–13.24)	<0.001	3.73 (1.57–8.90)	0.003
Regional LNM				
Para-aortic LNM	6.10 (4.08–9.10)	<0.001	1.98 (1.12–3.49)	0.018
Pelvic LNM	3.07 (2.20–4.28)	<0.001	1.65 (1.07–2.52)	0.022
Common iliac LNM	5.46 (3.69–8.07)	<0.001	1.75 (0.991–3.08)	0.054
Bilateral pelvic LNM	3.62 (2.57–5.12)	<0.001		
Bilateral common iliac LNM	7.25 (3.91–13.46)	<0.001	0.50 (0.22–1.13)	0.095
Number of pelvic MLNs (≥ 4)	8.41 (5.50–12.86)	<0.001	3.04 (1.56–5.91)	0.001
Diameter of pelvic MLNs (≥ 2cm)	4.12 (2.70–6.27)	<0.001		

Abbreviations: CI = confidence interval; FIGO = International Federation of Gynecology and Obstetrics; HR = hazard ratio; LNM = lymph nodes metastases; MLNs = metastatic lymph nodes; non-SCC = non-squamous cell carcinoma; SCC = squamous cell carcinoma.

**Table 3:** Univariate and multivariate analysis for disease-free survival.

Variables	Univariate analysis		Multivariate analysis	
	HR (95%CI)	p	HR (95%CI)	P
Histology				
SCC	Reference		Reference	
Non-SCC	2.14 (1.50–3.06)	<0.001	2.24 (1.55–3.23)	<0.001
FIGO stage				
IB	Reference		Reference	
IIA, IIB	2.57 (1.35–4.88)	0.004	2.11 (1.11–4.02)	0.023
IIIA, IIIB, IVA	4.66 (2.38–9.12)	<0.001	3.39 (1.72–6.71)	<0.001
Regional LNM				
Para-aortic LNM	5.09 (3.56–7.29)	<0.001	1.97 (1.22–3.20)	0.006
Pelvic LNM	2.80 (2.13–3.69)	<0.001	1.99 (1.44–2.74)	<0.001
Common iliac LNM	4.05 (2.83–5.79)	<0.001		
Bilateral pelvic LNM	3.12 (2.33–4.19)	<0.001		
Bilateral common iliac LNM	5.54 (3.08–9.95)	<0.001		
Number of pelvic MLNs (≥ 4)	5.41 (3.62–8.09)	<0.001	1.86 (1.10–3.13)	0.020
Diameter of pelvic MLNs (≥ 2cm)	3.05 (2.41–5.10)	<0.001		

Abbreviations: CI = confidence interval; FIGO = International Federation of Gynecology and Obstetrics; HR = hazard ratio; LNM = lymph nodes metastases; MLNs = metastatic lymph nodes; non-SCC = non-squamous cell carcinoma; SCC = squamous cell carcinoma.

**Table 4:** Univariate and multivariate analysis for local control.

Variables	Univariate analysis		Multivariate analysis	
	HR (95%CI)	P	HR (95%CI)	P
Histology				
SCC	Reference		Reference	
Non-SCC	2.31 (1.42–3.78)	0.001	2.18 (1.31–3.60)	0.003
FIGO stage				
IB	Reference		Reference	
IIA, IIB	3.14 (1.15–8.62)	0.026	2.50 (0.91–6.89)	0.076
IIIA, IIIB, IVA	5.19 (1.82–14.80)	0.002	3.44 (1.19–9.92)	0.023
Regional LNM				
Para-aortic LNM	6.66 (4.22–10.51)	<0.001	3.05 (1.75–5.34)	<0.001
Pelvic LNM	3.12 (2.11–4.63)	<0.001	1.77 (1.08–2.91)	0.025
Common iliac LNM	3.41 (2.05–5.70)	<0.001		
Bilateral pelvic LNM	3.85 (2.58–5.76)	<0.001		
Bilateral common iliac LNM	6.65 (3.22–13.75)	<0.001		
Number of pelvic MLNs (≥ 4)	5.78 (3.41–9.80)	<0.001		
Diameter of pelvic MLNs (≥ 2 cm)	4.66 (2.90–7.46)	<0.001	1.81 (1.02–3.20)	0.043

Abbreviations: CI = confidence interval; FIGO = International Federation of Gynecology and Obstetrics; HR = hazard ratio; LNM = lymph nodes metastases; MLNs = metastatic lymph nodes; non-SCC = non-squamous cell carcinoma; SCC = squamous cell carcinoma.

**Table 5:** Univariate and multivariate analysis for distant metastasis-free.

Variables	Univariate analysis		Multivariate analysis	
	HR (95%CI)	P	HR (95%CI)	p
Histology				
SCC	Reference		Reference	
Non-SCC	2.03 (1.27–3.26)	0.003	2.33 (1.45–3.75)	<0.001
FIGO stage				
IB	Reference		Reference	
IIA, IIB	2.14 (0.99–4.63)	0.054	1.78 (0.82–3.86)	0.147
IIIA, IIIB, IVA	3.45 (1.52–7.83)	0.003	2.59 (1.13–5.97)	0.025
Regional LNM				
Para-aortic LNM	3.46 (2.06–5.79)	<0.001		
Pelvic LNM	2.60 (1.81–3.72)	<0.001	2.09 (1.40–3.12)	<0.001
Common iliac LNM	3.88 (2.42–6.22)	<0.001		
Bilateral pelvic LNM	2.63 (1.77–3.90)	<0.001		
Bilateral common iliac LNM	4.09 (1.79–9.32)	0.001		
Number of pelvic MLNs (≥ 4)	4.90 (2.88–8.33)	<0.001	2.66 (1.48–4.79)	0.001
Diameter of pelvic MLNs (≥ 2 cm)	2.59 (1.53–4.38)	<0.001		

Abbreviations: CI = confidence interval; FIGO = International Federation of Gynecology and Obstetrics; HR = hazard ratio; LNM = lymph nodes metastases; MLNs = metastatic lymph nodes; non-SCC = non-squamous cell carcinoma; SCC = squamous cell carcinoma.

**Fig 2:**
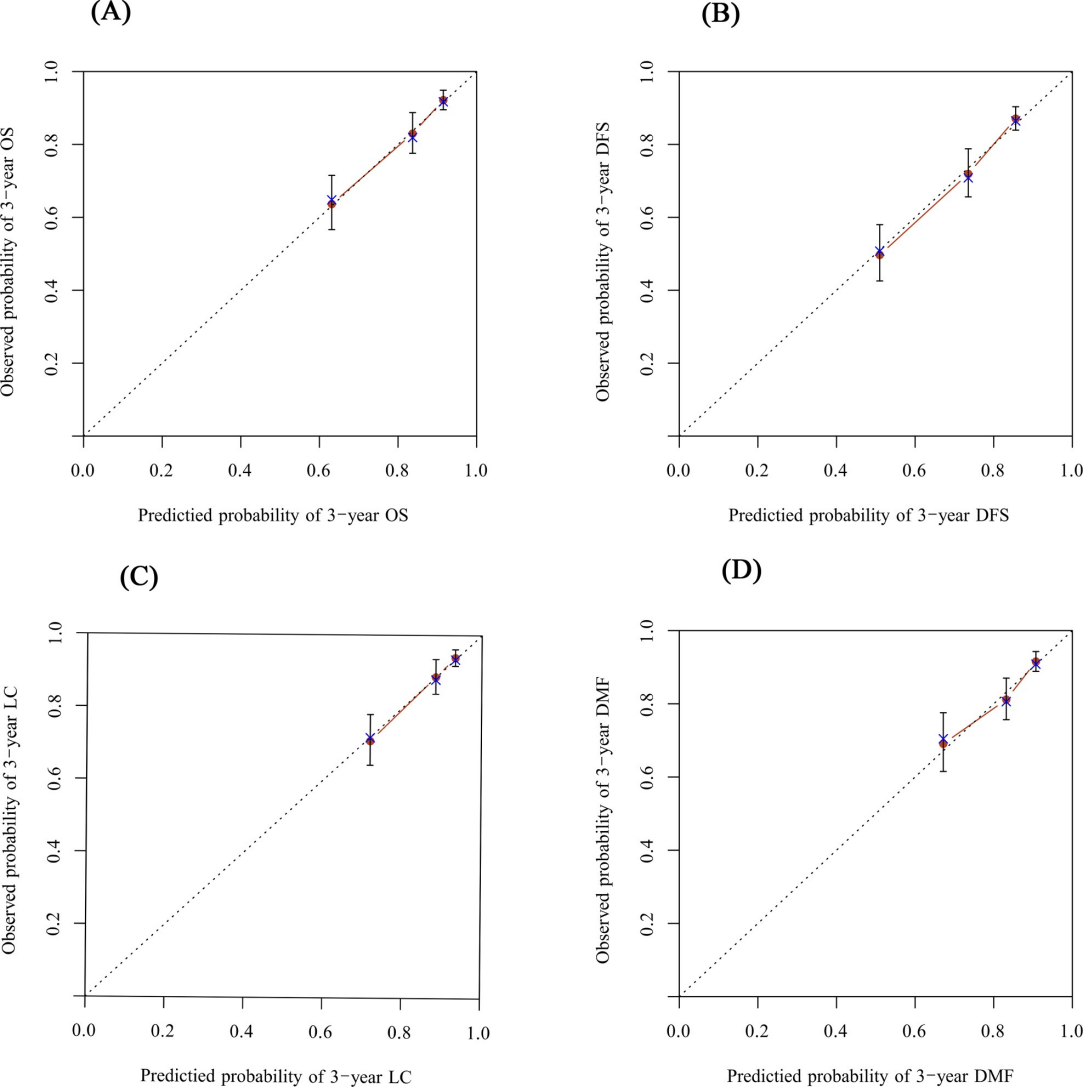
**Calibration curve for nomogram of the 3-year overall survival (OS, Fig 2A), disease-free survival (DFS, Fig 2B), local control (LC, Fig 2C) and distant metastasis-free (DMF, Fig 2D) prediction**.

The c-index was 0.73 for the nomogram predicting OS, which means a 73% chance that, given two randomly selected patients, the patient who died first had the worst predicted outcome. The c-index of the nomogram predicting DFS, LC and DMF were 0.71, 0.73 and 0.67, respectively. Fig 2 illustrates the calibration of the nomogram predicting 3-year OS (Fig 2A), DFS (Fig 2B), LC (Fig 2C) and DMF (Fig 2D), respectively. The dash line (45°line) represents the performance of an ideal nomogram, which predicted 100% survival and matched the actual survival. The solid line with dots represents constructed nomogram predicted probabilities with 95% confidence intervals. As shown in Fig 2A, 2B, 2C and 2D, the predicted probalility curves lay close to the ideal line.

The dash line (45°line) indicates the performance of an ideal nomogram for 3-year survival; solid line, performance of the current nomogram; filled dots, subcohort of present database; check marks, bootstrap-corrected predictions of the nomogram; vertical bar, 95% confidence interval.

## Discussion

In the present study, we focused on regional LNM, which were subclassified with lymph node regions (para-aortic, pelvic, and common iliac), as ipsilateral/bilateral (bilateral pelvic LNM and bilateral common iliac LNM), number (number of pelvic MLNs), and diameter (diameter of pelvic MLNs). All these variables were significant in univariate analysis of OS, DFS, LC and DMF. In addition, lymph node regions (para-aortic LNM and pelvic LNM), number of pelvic MLNs, and diameter of pelvic MLNs remained significant after multivariate analysis. Pelvic LNM has been well established as a prognostic factor of survival [7, 9]. The prognosis of patients with para-aortic LNM was poor after CCRT, with a DFS rate of 19.4% to 42% [17–19], which was much worse than patients with pelvic LNM. Our study showed that para-aortic LNM was an independent prognostic factor of OS, DFS and LC. However, the presence of para-aortic LNM was not incorporated in most previous nomograms [7, 10, 11], and this would influence the predictive abilities of these models. Among ipsilateral/bilateral, number, and diameter of involved pelvic lymph nodes, the number of pelvic MLNs ≥ 4 proved to be a significant factor of OS (hazard ratio [HR] 1.983, p = 0.011), DFS (HR 3.04, p = 0.001) and DMF (HR 2.66, p = 0.001), and diameter of pelvic MLNs was a independent factor of LC (HR = 1.81, p = 0.043). Reports on the association between number of pelvic MLNs and survival were limited. Li et al reported that an MLN number ≥ 3 was a independent prognostic factor of distant metastasis-free survival (HR 2.479, p = 0.026)[20]. In the future, if subclassification of N-stage tumor was considered by the AJCC, these 3 variables of para-aortic LNM, pelvic LNM, and number of pelvic MLNs could be take into account.

After CCRT, a considerable proportion of patients experienced treatment failure [1, 4, 5]. To improve survival, some new therapeutic strategies, such as adjuvant chemotherapy[21], neoadjuvant chemotherapy (INTERPLACE trial, NCT01566240), neoadjuvant chemotherapy followed by surgery[22], or extended-field radiotherapy[23] have been evaluated in clinical trials in recent years. It was reported that, compared with standard cisplatin based CCRT, gemcitabine plus cisplatin CCRT followed by adjuvant gemcitabine and cisplatin chemotherapy could improve OS, progression-free survival and decrease distant failure rates for patients with cervical cancer. The distant failure rates in standard CCRT group and CCRT plus adjuvant chemotherapy group were 16.4% and 8.1%, respectively (p = 0.005)[21]. Patients with cervical cancer may benefit from these new strategies. However, not all patients bear a high-risk of treatment failure. Using nomograms with high predictive abilities, patients could be stratified into different groups. For patients with high-risk of distant failure, adjuvant chemotherapy or neoadjuvant chemotherapy can be strategies to decrease distant failure rate and improve survival. For patients with high-risk of local recurrence, we can deliver higher radiation dose to the tumor. One the other hand, nomograms can be used in clinical trials on new therapeutic strategies. With the guidance of nomograms, the number of patients required to detect differnences in clinical trials may be reduced.

Although the number of patients were were comparatively large in the present study, patients were not divided into training group and validation group. It was recommended that, when the outcome is OS, the number of deaths should be greater than 10 times the number of predictors, so that the expected error in the predicted probabilities from the Cox model is less than 10%[16, 24]. In the present study, there were nine predictors in multivariate analysis and the number of events for OS, DFS, local control (LC) and distant metastasis-free (DMF) were 140, 205, 100 and 121, respectively. If we separated the patients into two groups (training group and validation group) at a ratio of 1:1 or 2:1, the number of events for OS, LC and DMF may be less than 90 in training cohort. Therefore, patients were not divided into training group and validation group.

There were some limitations in this study. Firstly, regional LNM was diagnosed by imaging modalities, including PET/CT, MRI, and CT. It has been reported that the sensitivity and specificity for MLNs of cervical cancer were 66%-82% [25, 26] and 95%-97% [25, 26] with PET/CT, 54%-86% [25–27] and 84%-93% [25–27]with MRI, and 50%-57% [25, 26] and 91%-92% [25, 26] with CT. With the comparatively low sensitivity of PET/CT, MRI, and CT, we would miss some occult MLNs compared with lymphadenectomy. Secondly, the nomograms were derived from patients in a single institute, and external validation was not performed to determine their generalizability. The nomograms should be externally validated before clinical use. Thirdly, the median follow-up in the present study is just 36.4 months, which is comparatively short. The main reason is that most patients were treated in recent years (2013 and 2014). And 5-year survival rates derived from the nomograms should be interpreted with caution.

## Conclusion

We constructed nomograms predicting OS and DFS based on histology, FIGO stage, para-aortic LNM, pelvic LNM and number of pelvic MLNs in patients with cervical cancer treated with concurrent chemoradiotherapy. The nomogram should be externally validated before clinical use.
